# Photocatalytic Activities of FeNbO_4_/NH_2_-MIL-125(Ti) Composites toward the Cycloaddition of CO_2_ to Propylene Oxide

**DOI:** 10.3390/molecules26061693

**Published:** 2021-03-17

**Authors:** Salwa Hussein Ahmed, Maram Bakiro, Ahmed Alzamly

**Affiliations:** Department of Chemistry, UAE University, Al-Ain 15551, United Arab Emirates; 201350201@uaeu.ac.ae (S.H.A.); maram_y@uaeu.ac.ae (M.B.)

**Keywords:** photocatalyst, band gap, FeNbO_4_/NH_2_-MIL-125(Ti), composite, cyclic carbonate

## Abstract

Photocatalytic utilization of CO_2_ in the production of value-added chemicals has presented a recent green alternative for CO_2_ fixation. In this regard, three FeNbO_4_/NH_2_-MIL-125(Ti) composites of different mole ratios were synthesized, characterized using Powder X-ray diffraction (PXRD), UV–vis diffuse reflectance spectroscopy (UV-Vis DRS), Brunauer–Emmett–Teller (BET), Scanning Electron Microscopy (SEM) and Energy Dispersive X-ray (EDX). PXRD patterns confirm the co-existence of the parent components in the prepared composites. Moreover, the surface area increased as the mole percent of NH_2_-MIL-125(Ti) in the composites increased due to the large surface area of NH_2_-MIL-125(Ti). Prepared composites were investigated for the photocatalytic insertion of CO_2_ into propylene oxide. FeNbO_4(75%)_/NH_2_-MIL-125(Ti)_(25%)_ showed the highest percent yield of 52% compared to the other two composites. Results demonstrate the cooperative mechanism between FeNbO_4_ and NH_2_-MIL-125(Ti) and that the reaction proceeded photocatalytically.

## 1. Introduction

Metal organic frameworks (MOFs) are microporous solid materials that have been developed over the last two decades. Constructed form inorganic nodes and multifunctional organic linkers, MOFs have outstanding potential applications in many fields due to their tremendously high surface area [1,2], tunable pore/channels size [3,4], gas adsorption capabilities [5,6] and storage capacity [7,8]. Among targeted applications, MOFs have interesting photocatalytic activities such as the photodegradation of various pollutants [9], water splitting [10], photoreduction of CO_2_ [11] and the photocatalytic production of value-added chemicals [12]. Particularly, MIL-125(Ti) has exhibited remarkable light harnessing capabilities that can trigger photocatalytic chemical reactions [13]. Nonetheless, MIL-125(Ti) has a large band gap value of 3.57 eV [14] which allows it to utilize light in the UV region [15]. Therefore, many methods were implemented to narrow its band gap to allow it to be utilized in the visible region. Such methods include introducing an amine functionality to the MOF linkers connecting the nodes [16]. NH_2_-MIL-125(Ti) possesses an optical band gap of 2.72 eV, where the presence of the amine functionality on the benzene ring of the linker donates electrons from its 2p orbital causing a red shift above the VB edge of MIL-125(Ti) [14]. Fu et al. investigated the first photoreduction of carbon dioxide to formate facilitated by visible light illumination using NH_2_-MIL-125(Ti) and triethanolamine as an electron donor [16]. Furthermore, Horiuchi et al. reported photocatalytic hydrogen production using NH_2_-MIL-125(Ti) in presence of the Pt nanoparticles co-catalyst under irradiation of visible light [17]. Additionally, Abdelhameed et al. investigated the post-synthetic modification of NH_2_-MIL-125(Ti) with Cr(III) and Ag nanoparticles for the removal of methylene blue (MB) under simulated day light exposure [18]. 

Metal and mixed metal oxides have relatively low surface areas, therefore the construction of MOF/mixed metal oxide composites provide composites with a higher surface area, while allowing each component to maintain its own integrity, contributing its characteristics to the new composite [19]. In this regard, Zhu et al. synthesized a BiOBr/NH_2_-MIL-125(Ti) composite and used it for the photocatalytic decomposition of rhodamine B (RhB) dye [20]. The composite showed a higher surface area compared to pure BiOBr and higher photocatalytic activity which was attributed to the transfer of the electron from Ti^4+^ to Ti^3+^ and the synergistic effect between BiOBr and the NH_2_-MIL-125(Ti) [20]. Likewise, Hu et al. reported the synthesis of BiOCl/NH_2_-MIL-125(Ti) composite which exhibited a large surface area allowing for more adsorption of contaminants such as tetracycline hydrochloride (TC), bisphenol A (BPA) and other reactive species [21]. Consequently, the composite showed improved photocatalytic activity towards the removal of TC and BPA. Likewise, superior photodegradation of methylene blue (MB) was demonstrated for the prepared series of BiOI/NH_2_-MIL-125(Ti) composites under visible light irradiation studied by Han and co-workers [22]. Recently, a BiNbO_4_/NH_2_-MIL-125(Ti) composite was prepared and used for the photocatalytic cycloaddition of CO_2_ to propylene oxide. The BiNbO_4_/NH_2_-MIL-125_(50:50)_ hybrid exhibited the highest conversion rate of 74% compared to pure BiNbO_4_ and NH_2_-MIL-125 [23]. In addition, Abdelhameed et al. further tuned the NH_2_-MIL-125(Ti) band gap by constructing a Ag_3_PO_4_/NH_2_-MIL-125(Ti) heterojunction composite [18]. The prepared Ag_3_PO_4_/NH_2_-MIL-125(Ti) composite showed an optical band gap shift of 2.39 eV which is noticeably smaller compared to that of pure NH_2_-MIL-125(Ti) (2.51 eV). The prepared composite showed higher photocatalytic activity for the removal of MB and RhB under visible light irradiation [18].

Iron niobate, FeNbO_4_, was investigated for a variety of applications including sensing [24], catalysis [25], and as photoanode material [26]. Nonetheless, limited research was conducted on the photocatalytic behavior of FeNbO_4_. Cho et al. investigated the photocatalytic removal of RhB dye using a narrow band gap FeNbO_4_ photocatalyst [27]. The hydrothermally prepared photocatalyst exhibited higher photocatalytic activity under tungsten halogen lamp irradiation which attributed to smaller particle size and fewer surface defects. Zhang et al. prepared FeNbO_4_ through a solid-state method and calcined it at different temperatures and used it for the photocatalytic degradation of methyl orange (MO) [28]. At a calcination temperature above 700 °C, pure FeNbO_4_ was formed, however, below this temperature, FeNb_2_O_6_ also formed. FeNbO_4_ along with the new formed phase of FeNb_2_O_6_ showed to increase the activity toward the removal of MO.

FeNbO_4_ was further investigated by incorporating rGO as a support material to improve the photocatalytic performance due to its high surface area and high electron mobility. Three different mass ratio FeNbO_4_/rGO composites were prepared for the photocatalytic cycloaddition of CO_2_ into propylene oxide under visible light irradiation [29]. As indicated, increasing rGO content in the composite increases the photocatalytic performance of the composite, where the FeNbO_4_-5%rGO composite showed the highest yield percent of 57%. Jones et al. investigated the photocatalytic performance of the FeNbO_4_/rGO composite for the degradation of norfloxacin under visible light irradiation using a 250 W Xe lamp [30]. The composite exhibited higher activity compared to pure FeNbO_4_ which is attributed to the ability of rGO to act as electron acceptor reducing the recombination of the photogenerated electron-hole pairs. 

In this report, FeNbO_4_/NH_2_-MIL-125(Ti) composites with different mole ratios of FeNbO_4_ added were prepared for the first time. Composites were characterized using different analytical techniques: PXRD, UV-Vis DRS, BET, SEM and EDX. Moreover, their photocatalytic performance was evaluated toward the cycloaddition of CO_2_ to propylene oxide. 

## 2. Experimental

### 2.1. Materials

Iron (III) nitrate (Fe(NO_3_)_3_·9H_2_O), Ammonium niobate (V) oxalate (C_4_H_4_NNbO_9_·_X_H_2_O), ammonium hydroxide (NH_4_OH), barium sulfate (BaSO_4_), chloroform-d (CDCl_3_), methylene chloride (CH_2_Cl_2_, DCM), dimethylformamide (C_3_H_7_NO, DMF), 2-amino benzene dicarboxylic acid (C_8_H_7_NO_4_), titanium isopropoxide (C_12_H_28_O_4_Ti), methanol (CH_3_OH) tetra-n-butylammonium bromide (C_16_H_36_BrN, TBAB), acetonitrile (CH_3_CN) and propylene oxide (C_3_H_6_O, PO) were obtained from Sigma Aldrich and used without purification. 

### 2.2. Synthesis of Pure FeNbO_4_

Co-precipitation approach was implemented for the synthesis of FeNbO_4_. In a typical procedure: 0.015 mol of Ferric nitrate nonahydrate in 10 mL of DI water and 0.015 mol of ammonium niobate (V) oxalate was dissolved in 70 mL of DI water. The Ferric solution was then added dropwise to the Nb solution, immediately, the mixture turned yellow. The mixture left under vigorous stirring, and the pH was adjusted to 2 by the addition of NH_4_OH. Reaction mixture allowed to stir overnight. A water bath was used to evaporate excess solvent. The solid product was then allowed to dry in an oven at 100 °C. The solid was then calcinated at 1100 °C for 6 h.

### 2.3. Synthesis of NH_2_-MIL-125(Ti)

NH_2_-MIL-125(Ti) was synthesized by dissolving 1 g (6 mmol) of 2-amino benzene dicarboxylic acid in a mixture of 50 mL DMF and 50 mL methanol. The mixture was placed in a 100 mL sealed tube and 910 μL (3 mmol) of titanium isopropoxide was added, a yellow powder was formed immediately. The sealed tube was heated at 130 °C for 1 day. The produced powder was filtered and washed with DMF to separate unreacted materials. The product was washed several times with methanol.

### 2.4. Synthesis of FeNbO_4_/NH_2_-MIL-125(Ti) Composites

Three different mole ratios of FeNbO_4_/NH_2_-MIL-125(Ti) were prepared. FeNbO_4 (25%)_/NH_2_-MIL-125(Ti)_(75%)_ composite was prepared by the simple mixing of 25 mol% of FeNbO_4_ and 75 mol% of NH_2_-MIL-125(Ti) in 5 mL acetone. The other two composites, FeNbO_4(50%)_/NH_2_-MIL-125(Ti)_(50%)_ and FeNbO_4(75%)_/NH_2_-MIL-125(Ti)_(25%),_ were prepared following the above-described procedure.

## 3. Characterization 

### 3.1. Powder X-ray Diffraction Spectroscopy (PXRD)

Powder X-ray diffraction were recorded on Shimadzu-6100 X-ray powder instrument. The X-ray tube voltage was 40 kV, current 30 mA and wavelength λ of 1.542 Å. Samples were scanned within the range 10°–60° at a rate of 2°/min.

### 3.2. UV-Vis Diffuse Reflectance Spectroscopy

Band gaps were measured using Shimadzu UV-3600 UV-Vis spectrometer operates over a range of 200 nm to 800 nm. Tauc plots method [31] was used for calculating the band gap energies for the prepared photocatalysts. The band gaps were calculated applying Equation (1) where α, *h*, and *v* are the absorption coefficient, Planck’s constant and light frequency, respectively. The *n* value is characteristic of the semiconductor transition (*n* = 1/2 for direct allowed transition), A is a constant and $E_{g}$ is the measured band gap [32].

(1) $${\left(\alpha h\nu \right)}^{\frac{1}{n}}=A\left(h\nu -E_{g}\right)$$

The band gap energy $\left(E_{g}\right)$ was calculated by plotting (α*hv*${)}^{2\;}$ vs. *hv* and extrapolating the linear region to the point that the linear line intersects with the *hv* axis [33].

### 3.3. Scanning Electron Microscopy (SEM) and Energy-Dispersive X-ray Spectroscopy (EDX) 

Surface morphology and elemental composition were analyzed using a FEI SEM Quattro S scanning electron microscope equipped with EDS-Oxford INCA PENTA runs at 30 KV. Prior to the analysis, each sample was positioned on a sample holder which was covered with a carbon tape followed by an overlay with gold. 

### 3.4. BET Surface Area and Porosity

Quantochrome Autosorb-1 volumetric gas sorption was used for BET surface area and porosity determination, the BET surface area was estimated using a nitrogen adsorption-desorption curve at 77 K. Prior to the analysis, each sample was degassed at 150 °C for 2 h. Brunauer–Emmett–Teller (BET) theory was used to calculate the surface area. Moreover, Barett–Joyner–Halenda (BJH) model was adopted in pore size determination.

### 3.5. Nuclear Magnetic Resonance (NMR)

For product identification, ^1^H and ^13^C NMR were recorded on a Varian-400 MHz using chloroform-d as a solvent.

### 3.6. Fourier Transform Infrared Spectroscopy (FT-IR)

For functional group identification, ATR-FTIR spectrum was recorded on a Fourier transform infrared spectrophotometer IR Prestige-21, Shimadzu operates in the range of 500 to 3500 cm^−1^.

## 4. Photocatalytic Activity

Photocatalytic activities of prepared composites were evaluated for the cycloaddition of CO_2_ to epoxide. Reaction mixture contained 100 μL propylene oxide, 1 mL methanol as electron hole scavenger, 4 mL acetonitrile, 9 mg TBAB as co-catalyst, 0.045 moL CO_2_ and 50 mg of the prepared photocatalyst. The round-bottom flask containing the mixture was kept 10 cm away from the light source (Halogen lamp-500 W) and allowed to stand for 72 h [34]. The reaction then was stopped and the photocatalyst was filtered and product was isolated from TBAB by separation using a mixture of water and DCM. The DCM layer was evaporated, and the product was isolated and characterized using FT-IR, ^1^HNMR, and ^13^CNMR.

## 5. Results and Discussion

### 5.1. PXRD Analyses of Pure FeNbO_4_ and FeNbO_4_/NH_2_-MIL-125(Ti) Composites 

**Figure 1:**
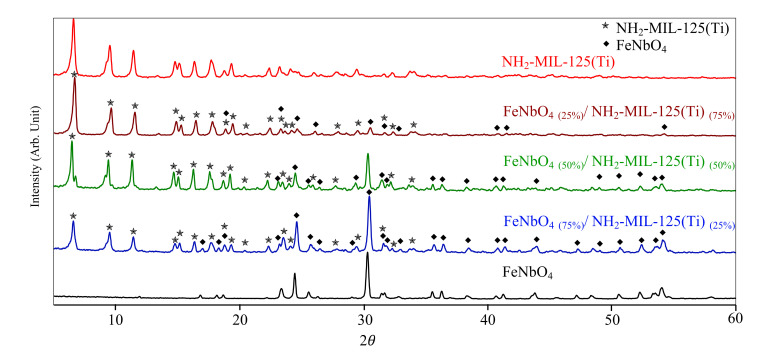
PXRD patterns of FeNbO_4_, NH_2_-MIL-125(Ti), and FeNbO_4_/NH_2_-MIL-125(Ti) composites.

All diffraction peaks of synthesized NH_2_-MIL-125(Ti) are consistent with the first NH_2_-MIL-125(Ti) reported by Fu et al. [16]. On the other hand, pure FeNbO_4_ diffraction pattern is indexed to the monoclinic phase (JCPDS file No.16-0374). Thus, PXRD patterns of prepared FeNbO_4_/NH_2_-MIL-125(Ti) composites display both diffraction peaks of pure NH_2_-MIL-125(Ti) and FeNbO_4_, implying an effective incorporation of both components in the newly formed composites. Notably, peak intensity represents the amount of the component present in the composite, e.g., in the FeNbO_4(75%)/_NH_2_-MIL-125(Ti)_(25%)_ composite, FeNbO_4_ peaks are more intense compared to NH_2_-MIL-125(Ti) peaks. Moreover, NH_2_-MIL-125(Ti) peak intensity increases as the mole ratio of NH_2_-MIL-125(Ti) increases in the composite as shown in Figure 1. The crystal size shown in Table 1 was calculated using Scherrer equation [35]. As expected, all prepared composites attained the same calculated crystal size of 38.13 nm.

### 5.2. UV-Vis DRS for Pure FeNbO_4_ and Composite Photocatalysts

**Figure 2:**
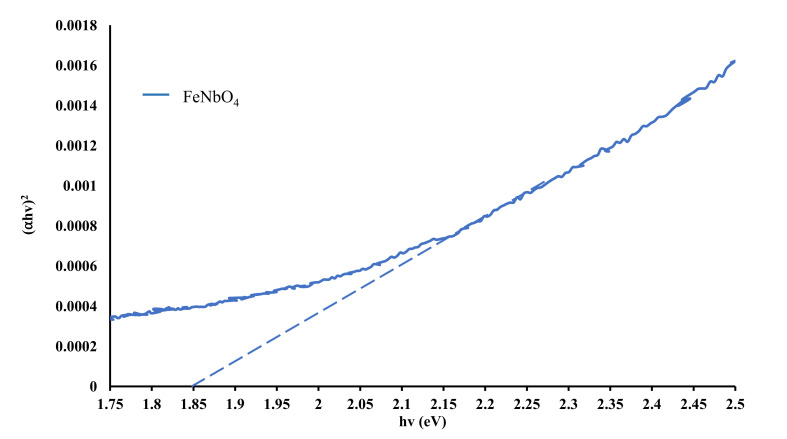
Tauc plot of pure FeNbO_4_.

**Figure 3:**
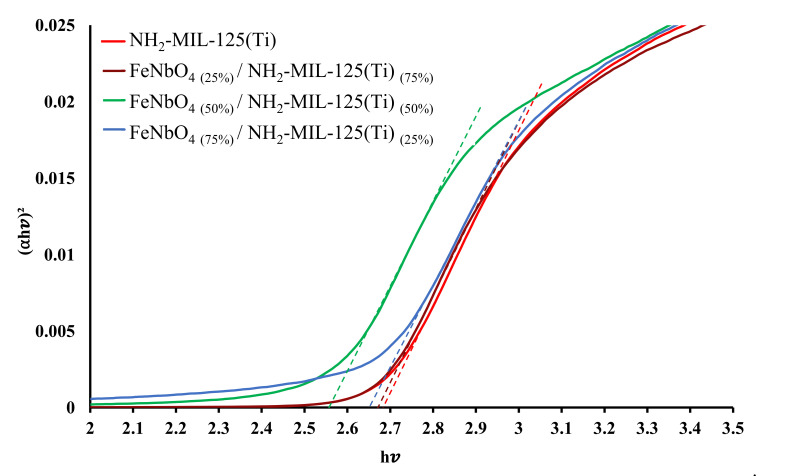
Tauc plot of FeNbO_4_ and NH_2_-MIL-125(Ti) and the prepared composites.

Optical absorptions were obtained for pure FeNbO_4_, NH_2_-MIL-125(Ti) and their corresponding composite photocatalysts. Band gaps were calculated using Tauc plot method and are presented in Figure 2 and Figure 3. NH_2_-MIL-125(Ti) displays the largest band gap of 2.68 eV, same as reported by Wang et al. [36]. Prepared FeNbO_4_/NH_2_-MIL-125(Ti) composites of different mole ratio exhibited a red shift corresponds to a narrower band gap. It was noted that, as FeNbO_4_ mole percent increases, the calculated band gap decreases as shown in Table 1. Interestingly, FeNbO_4(50%)_/NH_2_-MIL-125(Ti)_(50%)_ showed the lowest band gap at 2.55 eV. 

### 5.3. N_2_ Adsorption-Desorption Analyses for Pure FeNbO_4_ and Composites

**Figure 4:**
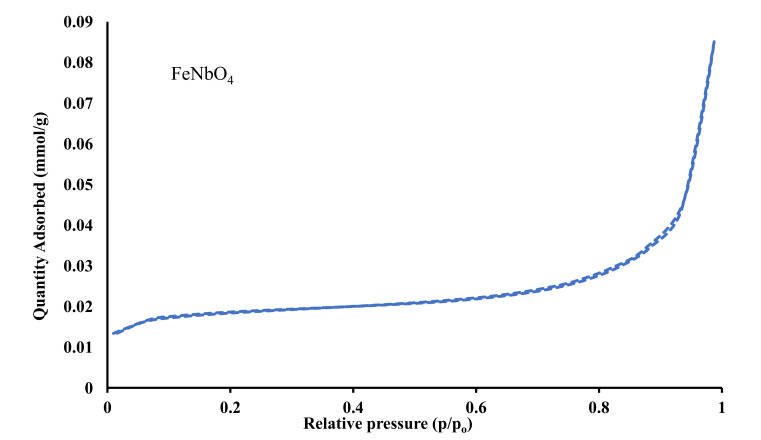
N_2_ adsorption-desorption isotherm of pure FeNbO_4_.

**Figure 5:**
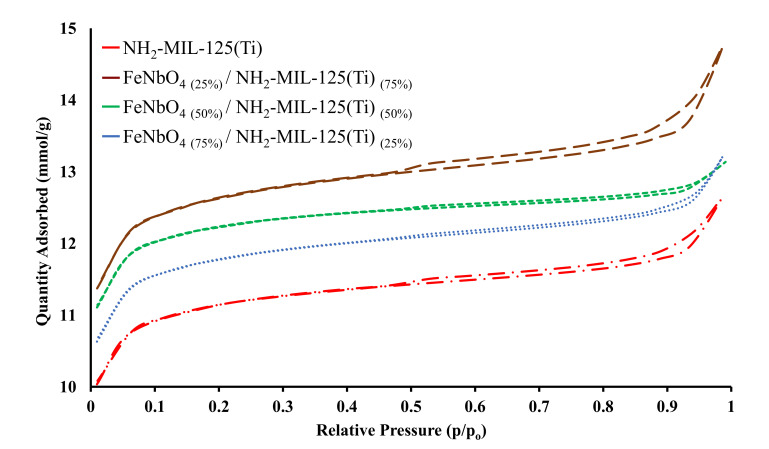
N_2_ adsorption-desorption isotherm of pure NH_2_-MIL-125(Ti) and the three different mole ratio composites of FeNbO_4_/NH_2_-MIL-125(Ti).

Surface area and porosity of pure NH_2_-MIL-125(Ti), FeNbO_4_, and the composite photocatalysts, were analyzed using N_2_ adsorption-desorption isotherms (Figure 4 and Figure 5). Pure NH_2_-MIL-125(Ti) exhibited type- IV isotherm characterized by a H3 hysteresis loop observed at a relative pressure range between 0.5 and 1.0 which is associated with capillary condensation indicating the existence of a mesoporous structure. The three different composites displayed type-IV isotherms with a hysteresis loop as that of pure NH_2_-MIL-125(Ti). The calculated surface area for NH_2_-MIL-125(Ti), FeNbO_4(25%)_/NH_2_-MIL-125(Ti)_(75%)_, FeNbO_4(50%)_/NH_2_-MIL-125(Ti)_(50%)_ and FeNbO_4(75%)_/NH_2_-MIL-125(Ti)_(25%)_ are 1025.71 m^2^/g, 856.40 m^2^/g, 820.92 m^2^/g and 666.13 m^2^/g, respectively. The surface area of the composites decreased as the mole ratio of FeNbO_4_ increased which is attributed to the small surface area of the prepared FeNbO_4_ (Table 1).

### 5.4. Scanning Electron Microscopy (SEM) and Energy-Dispersive X-ray Spectroscopy (EDX)

**Figure 6:**
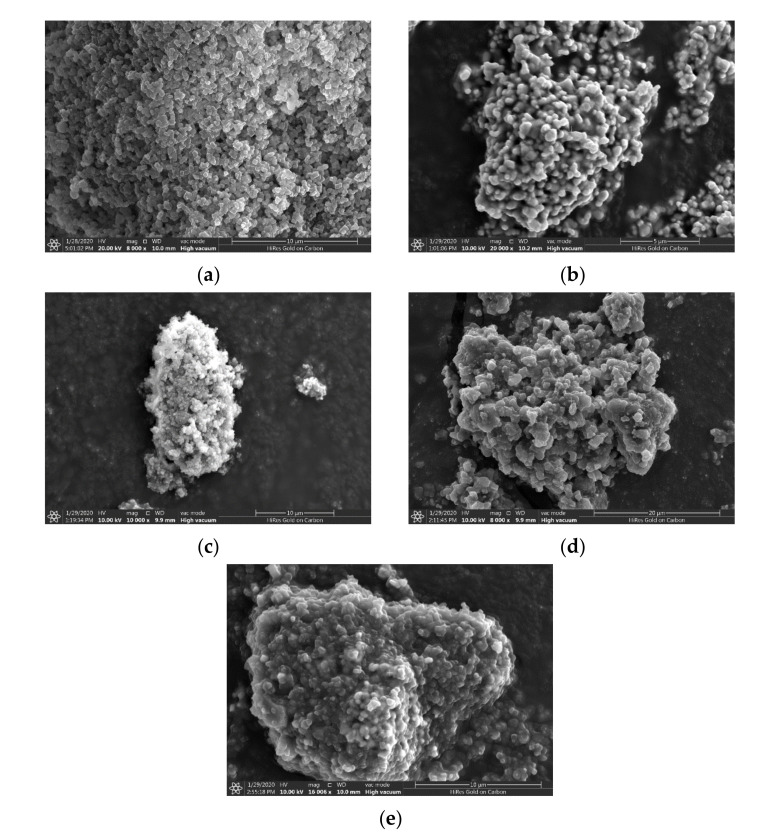
SEM images of (**a**) NH_2_-MIL-125(Ti), (**b**) FeNbO_4_, (**c**) FeNbO_4 (25%)_/NH_2_-MIL-125(Ti) _(75%)_, (**d**) FeNbO_4 (50%)_/NH_2_-MIL-125(Ti) _(50%)_ and (**e**) FeNbO_4 (75%)_/NH_2_-MIL-125(Ti) _(25%)_.

Elemental analyses and SEM images of pure NH_2_-MIL-125(Ti), FeNbO_4_ and their composite photocatalysts are presented in Figure 6 and Figure S1. NH_2_-MIL-125(Ti) shows a mixture of agglomerated spherical and cube-like structure with sharp edges, while FeNbO_4_ exhibited an agglomerated sphere-like morphology. As displayed in Figure 6c, FeNbO_4 (25%)_/NH_2_-MIL-125(Ti) _(75%)_ exhibited a similar morphology to that of pure NH_2_-MIL-125(Ti) with severe agglomeration and less defined edges. Notably, as percent FeNbO_4_ increases, agglomeration increases, and the morphology increasingly resembles that of pure FeNbO_4_. Accordingly, SEM images conclude that FeNbO_4_ is well integrated with NH_2_-MIL-125(Ti) which can assist in the transmission of the photogenerated electrons throughout the composites.

EDX analyses confirmed the elemental composition of pure NH_2_-MIL-125(Ti), FeNbO_4_, and composite photocatalysts with no extra peaks observed for any impurities present in the sample (Table 2). Elemental mapping shows the distribution of C, N, O, Ti, Fe, and Nb in the samples (Figure S2).

### 5.5. Photocatalytic Reaction of FeNbO_4_, NH_2_-MIL-125(Ti) and FeNbO_4_/NH_2_-MIL-125(Ti) Composite Photocatalysts

Selective Propylene carbonate formation was confirmed using ^1^H and ^13^C NMR and FT-IR. There were no extra peaks detected for any polymeric products, all peaks in the NMR spectra were assigned to only one product formation, i.e., selective propylene carbonate with the correct ratio of integration (see supporting information Figures S3 and S4). Moreover, the FT-IR graph illustrates the presence of C=O and C−O functional groups (Figure S5).

**Figure 7:**
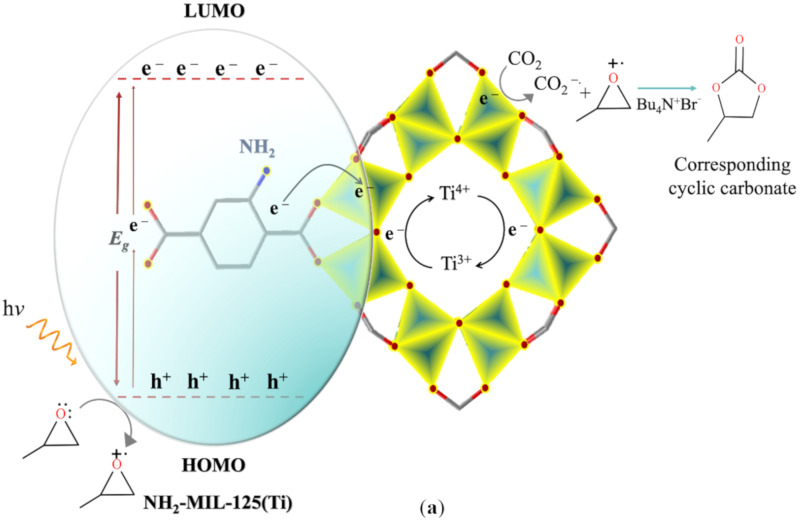

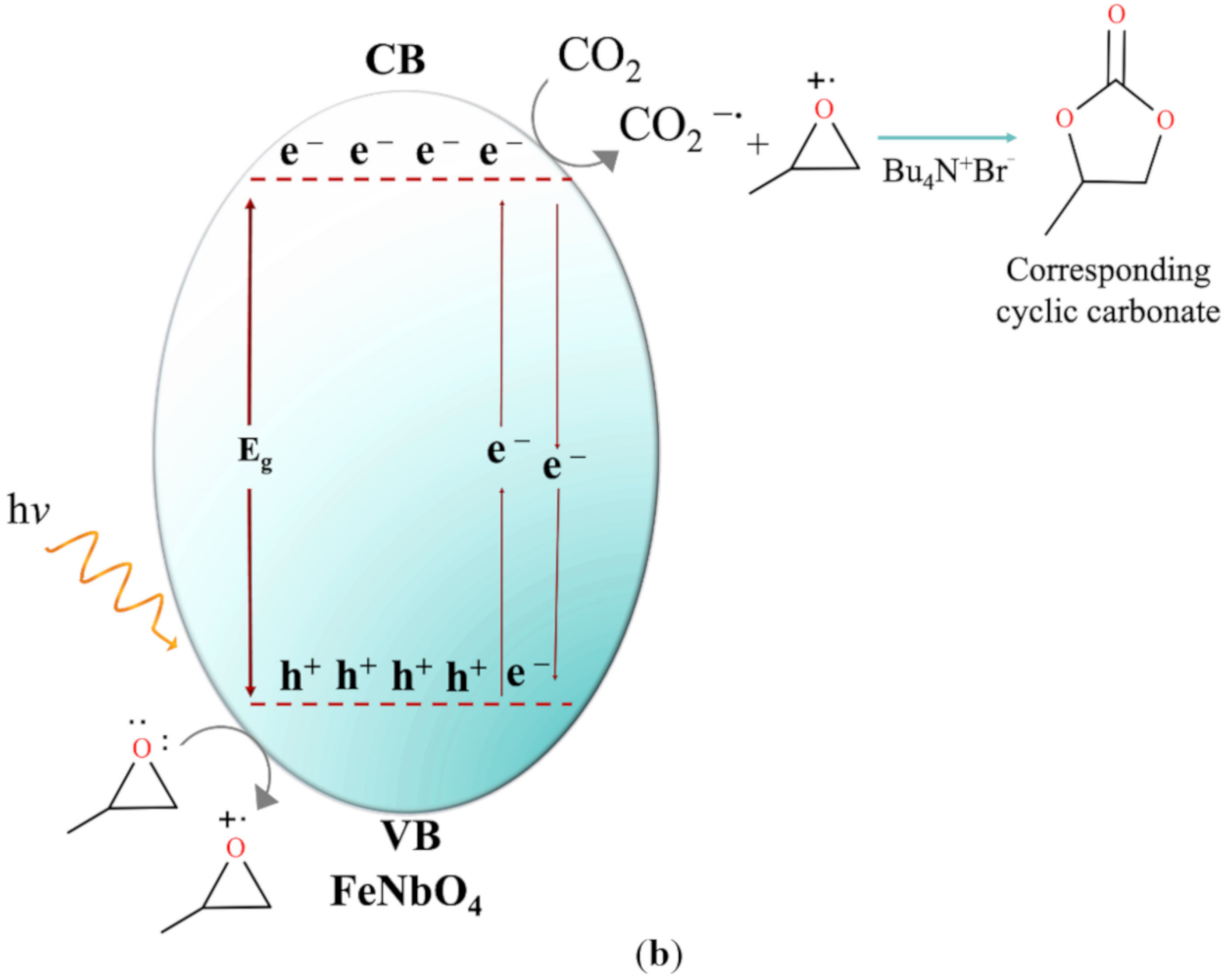
Proposed mechanism of (**a**) NH_2_-MIL-125(Ti) and (**b**) FeNbO_4_ for the photocatalytic addition of CO_2_ into propylene oxide.

**Figure 8:**
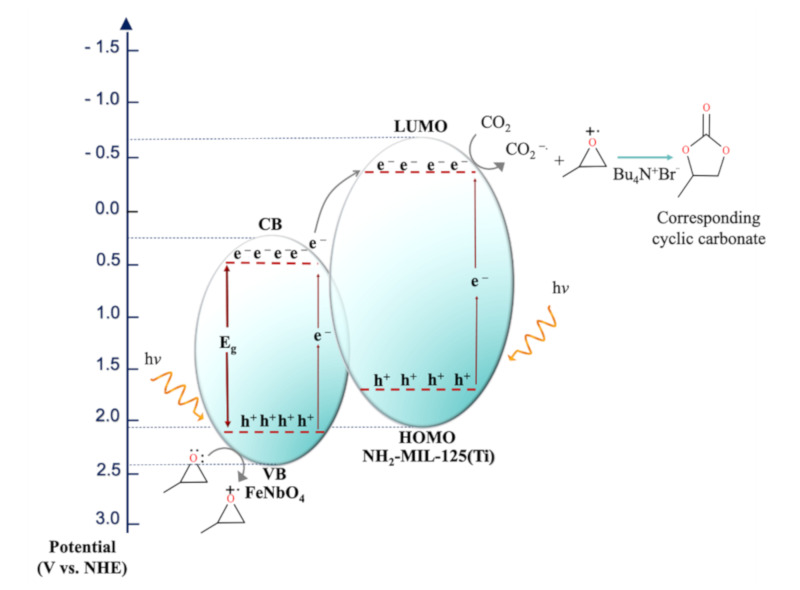
Proposed mechanism of FeNbO_4_/NH_2_-MIL-125(Ti) for the photocatalytic addition of CO_2_ into propylene oxide.

A low percent yield (12%) was observed for pure NH_2_-MIL-125(Ti) compared to pure FeNbO_4_ and their composites. It was proven that the NH_2_-MIL-125(Ti) photoreaction proceeds via the (LMCT) ligand to metal charge transfer [16]. Upon light irradiation, generated electrons from the linker are transformed to Ti^4+^ to facilitate its reduction to Ti^3+^ [37,38] while methanol acts as an electron donor (Figure 7a). Evidently, there are two competing processes involved; the first indicates much of the CO_2_ molecules were adsorbed into the NH_2_-MIL-125(Ti), the second involves the formation of a carbon dioxide radical anion which facilitates the cycloaddition to epoxide. Likewise, the FeNbO_4_ calculated percent yield was low (28%) which can be attributed to the high recombination rate, the mechanism for which is presented in Figure 7b. However, the photocatalytic efficiency was higher for the prepared composites [39,40,41] where a high yield was achieved as the FeNbO_4_ ratio increased in the composite. This can be related to the ability of FeNbO_4_ to absorb more visible light and excite more photogenerated electrons. NH_2_-MIL-125(Ti) serves as a CO_2_ adsorbent, therefore Ti^3+^ reduces CO_2_ to CO_2_^−^ followed by its addition to propylene oxide activated by TBAB. This electron transfer process suppresses the recombination rate of the generated e^−^ and h^+^ pairs. Methanol acts as a hole scavenger providing more electrons for the continuous reduction of Ti^4+^; Figure 8 outlines this mechanism as suggested by Prajapati et al. [42]. The potential of the conduction band (CB) and the valence band (VB) of FeNbO_4_ were calculated using the following equations [25]:

E_CB_ = 𝜒 − E_0_ − 0.5E_g_ (2)

E_VB_ = E_CB_ + E_g_ (3)

E_CB_ in Equation (2) refers to the potential of the CB in eV. χ is the Mulliken electronegativity geometric mean calculated for the constituent atoms of the semiconductor. E_0_ is ~4.5 eV which is the free electron on the H_2_ redox scale and E_g_ is the band gap. The valence band potential was calculated using Equation (3). FeNbO_4_ possesses a calculated conduction band potential of 0.64 V (vs. NHE), given that the calculated E_g_ of FeNbO_4_ is 1.85 eV as obtained from UV-vis DRS. The valence band potential was calculated to be 2.49 V. In addition, the band potential of the highest occupied molecular orbital (HOMO) and the lowest unoccupied molecular orbital (LUMO) of NH_2_-MIL-125(Ti) is −0.59 V and 2.09 V (vs, NHE), respectively. These values were calculated by Wang et al., for NH_2_-MIL-125(Ti) with E_g_ = 2.68 eV (the same value was obtained using UV-vis DRS for the prepared NH_2_-MIL-125(Ti) [36]. A blank experiment was conducted by adding TBAB (entry 6, Table 3) with no photocatalyst under visible light irradiation. The calculated yield was 15% which confirms the photocatalytic nature of this process. Further, reactions were conducted in the dark using synthesized photocatalysts without and with applying heat up to 75 °C. Only FeNbO_4 (75%)_/NH_2_-MIL-125(Ti) _(25%)_ composite photocatalyst showed low activity under these conditions. An insignificant yield was observed when no light and no heat involved (entry 7, Table 3), implying that the reaction is prominently photocatalytic in nature. On the other hand, applying heat to the reaction mixture increased the yield slightly (entry 8, Table 3) which suggests that heat influences the reaction. In fact, carbon dioxide utilization for cycloaddition of epoxides accomplished hydrothermally where temperature and pressure of CO_2_ have found to play a key role in cyclic carbonate formation [43]. However, this does not rule out that as entries 7 and 8 suggest that the reaction proceeds predominantly photocatalytic. It is worth mentioning that when trying to cycle the photocatalyst after the first run, the photocatalyst dissipates during filtration due to the fine powdery nature of the photocatalyst.

## 6. Conclusions

In conclusion, three composites were successfully prepared and characterized. PXRD patterns of the composites confirm the co-existence of both FeNbO_4_ and NH_2_-MIL-125(Ti). NH_2_-MIL-125(Ti) showed the highest surface area of 1025.71 m^2^/g, which decreased as the mole ratio of FeNbO_4_ increased due to the insignificant surface area of FeNbO_4_. FeNbO_4_/NH_2_-MIL-125(Ti) composites presented higher photocatalytic activity in comparison to the pure FeNbO_4_ and NH_2_-MIL-125(Ti), indicating that the formed heterojunction improves the photocatalytic activity of the materials due to synergistic effects between FeNbO_4_ and NH_2_-MIL-125(Ti). Variable reaction conditions were implemented to understand the role of the photocatalyst and visible light irradiation. The obtained results underline that the reaction proceeds predominantly photocatalytically.

## Figures and Tables

**Table 1:** Physical properties of NH_2_-MIL-125(Ti), FeNbO_4_ and their composites.

Samples	Band Gap (eV)	Crystal Size (nm)	BET Surface Area (m^2^/g)	Pore Volume (cm^3^/g)	Pore Diameter (Å)
NH_2_-MIL-125(Ti)	2.68	32.26	1025.71	0.57	22.35
FeNbO_4 (25%)_/NH_2_-MIL-125(Ti) _(75%)_	2.66	38.13	856.40	0.49	22.70
FeNbO_4 (50%)_/NH_2_-MIL-125(Ti) _(50%)_	2.55	38.13	820.92	0.45	21.75
FeNbO_4 (75%)_/NH_2_-MIL-125(Ti) _(25%)_	2.65	38.13	666.13	0.37	22.09
FeNbO_4_	1.85	49.06	1.34	0.0019	57.81

**Table 2:** Weight and atomic percentage of NH_2_-MIL-125(Ti), FeNbO_4_ and their composites.

Samples	Weight %						Atomic %					
	C	N	O	Ti	Fe	Nb	C	N	O	Ti	Fe	Nb
NH_2_-MIL-125(Ti)	22.78	5.54	25.83	45.86	-	-	39.00	8.13	33.19	19.68	-	-
FeNbO_4 (25%)_/NH_2_-MIL-125(Ti) _(75%)_	30.85	9.14	34.26	16.53	1.36	7.85	44.16	11.22	36.82	5.93	0.42	1.45
FeNbO_4 (50%)_/NH_2_-MIL-125(Ti) _(50%)_	20.72	4.96	31.44	30.74	0.45	11.69	35.79	7.35	40.77	13.31	0.17	2.61
FeNbO_4 (75%)_/NH_2_-MIL-125(Ti) _(25%)_	35.52	5.95	31.18	12.93	1.29	13.14	51.30	7.36	33.80	4.68	0.40	2.45

**Table 3:** Reaction conditions and results of NH_2_-MIL-125(Ti), FeNbO_4_ and their composites ^(a)^.

Entry	Photocatalyst	Reaction Conditions	Yield (%) ^(b)^
1	NH_2_-MIL-125(Ti)	visible light	12
2	FeNbO_4 (25%)_/NH_2_-MIL-125(Ti) _(75%)_	visible light	39
3	FeNbO_4 (50%)_/NH_2_-MIL-125(Ti) _(50%)_	visible light	30
4	FeNbO_4 (75%)_/NH_2_-MIL-125(Ti) _(25%)_	visible light	52
5	FeNbO_4_	visible light	28
6 ^(c)^	TBAB	visible light	15
7	FeNbO_4 (75%)_/NH_2_-MIL-125(Ti) _(25%)_	no light	2
8	FeNbO_4 (75%)_/NH_2_-MIL-125(Ti) _(25%)_	heat with no light ^(d)^	11

^(a)^ Reaction was conducted using propylene oxide (1.4 mmol), 4 mL CH_3_CN, 1 mL MeOH as hole scavenger, TBAB (0.028 mmol), 50 mg photocatalyst and 0.045 moL CO_2_, reaction conducted for 72 h using 500 W visible light halogen lamp. ^(b)^ Yield was calculated based on actual yield of product recovered. ^(c)^ Reaction run with no photocatalyst. ^(d)^ Reaction was conducted at temperature of 75 °C.

## Data Availability

Data of the compounds are available from the authors.

## Supplementary Materials

The following are available online, Figure S1: EDX for (a) NH_2_-MIL-125(Ti), (b) FeNbO_4_, (c) FeNbO_4 (25%)_/NH_2_-MIL-125(Ti) _(75%)_, (d) FeNbO_4 (50%)_/NH_2_-MIL-125(Ti) _(50%)_ and (e) FeNbO_4 (75%)_/NH_2_-MIL-125(Ti) _(25%)_., Figure S2: SEM elemental mapping of (a) NH_2_-MIL-125(Ti), (b) FeNbO_4_, (b) FeNbO_4 (25%)_/NH_2_-MIL-125(Ti) _(75%)_, (c) FeNbO_4 (50%)_/NH_2_-MIL-125(Ti) _(50%)_ and (d) FeNbO_4 (75%)_/NH_2_-MIL-125(Ti) _(25%)_, Figure S3: ^1^H NMR spectrum for obtained propylene carbonate, Figure S4: ^13^C NMR spectrum for obtained propylene carbonate, Figure S5: FTIR spectrum for obtained propylene carbonate.

## Sample Availability

Samples of the compounds are not available from the authors.
