# Supplementary material for: Photocatalytic Activities of FeNbO4/NH2-MIL-125(Ti) Composites toward the Cycloaddition of CO2 to Propylene Oxide
Source: Molecules. 2021 Mar 17;26(6):1693. doi: 10.3390/molecules26061693 (PMC8002832; doi:10.3390/molecules26061693)
Supplement: Supplementary file 1 [file molecules-26-01693-s001.zip › molecules-1149645-supplementary.pdf]

molecules

Photocatalytic Activity of $FeNbO_4$/NH_2-MIL-125(Ti) Composites Toward the Cycloaddition of CO_2 into Propylene Oxide

Salwa Hussein Ahmed[a], *Maram Bakiro*[a] *and Ahmed Alzamly*[a*],

[a] Department of Chemistry, UAE University, P.O. Box 15551, Al-Ain, UAE

*Corresponding author: ahmed.alzamly@uaeu.ac.ae

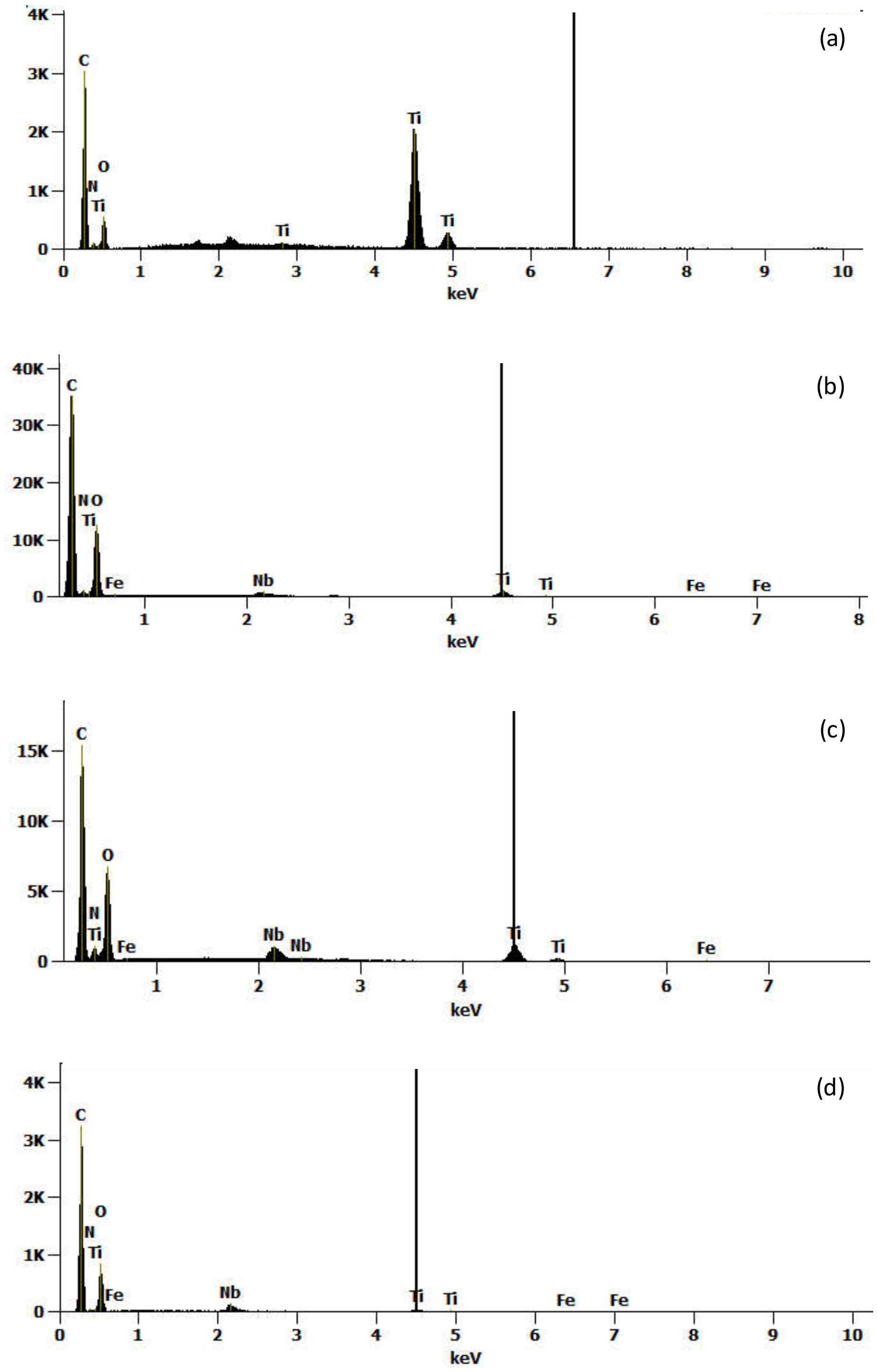

Figure S1. EDX for (a) NH_2-MIL-125(Ti), (b) $FeNbO_4$, (c) $FeNbO_4$ $_{(25\%)}$/NH_2-MIL-125(Ti) $_{(75\%)}$, (d) $FeNbO_4$ $_{(50\%)}$/NH_2-MIL-125(Ti) $_{(50\%)}$ and (e) $FeNbO_4$ $_{(75\%)}$/NH_2-MIL-125(Ti) $_{(25\%)}$.

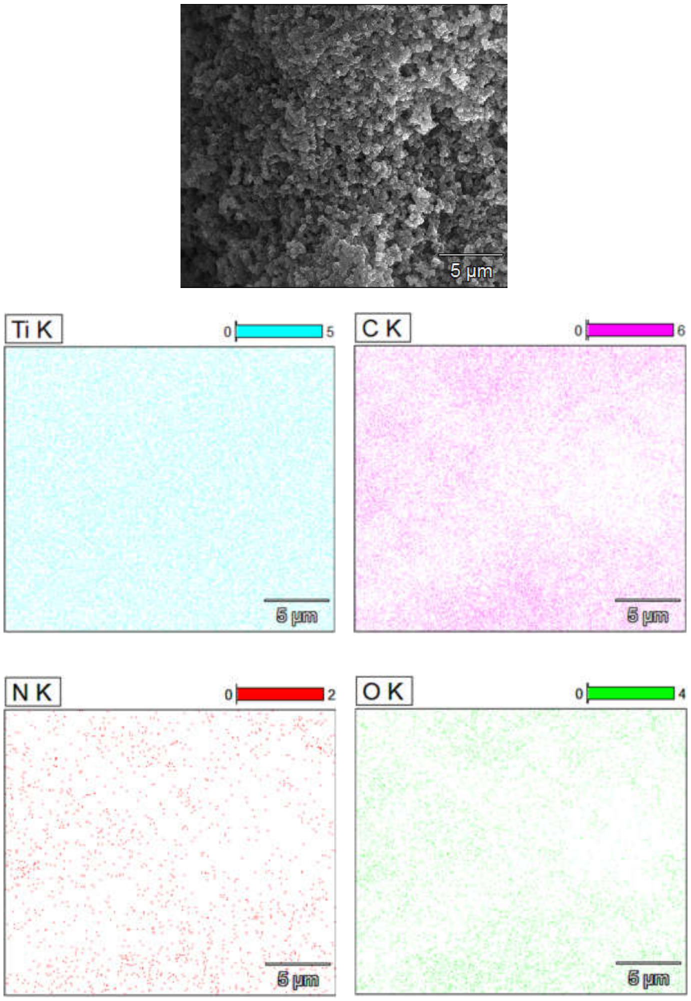

(a)

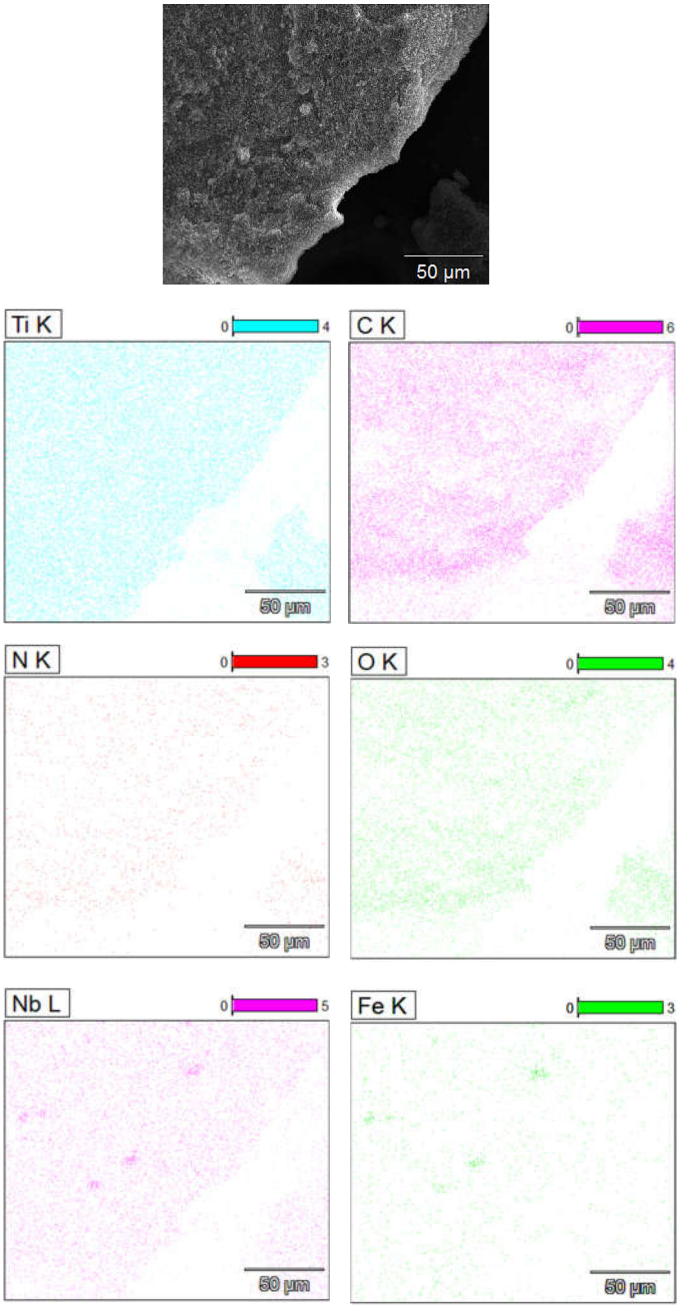

(b)

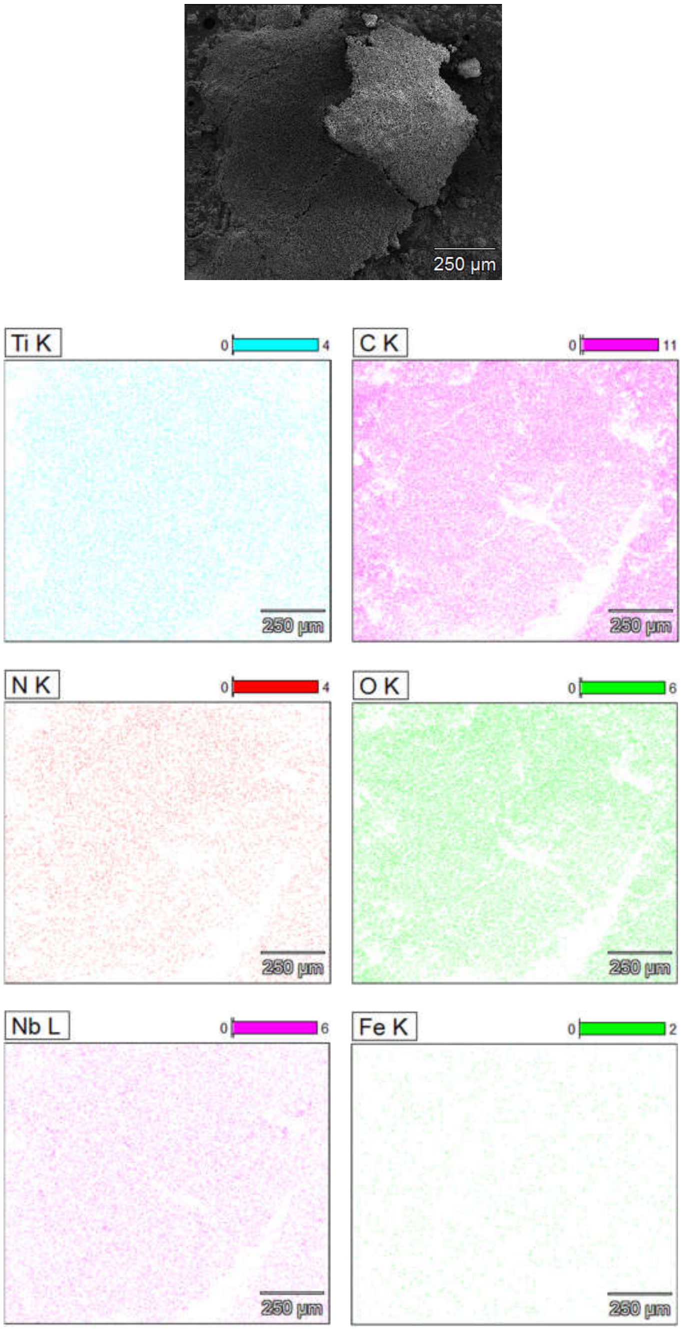

(c)

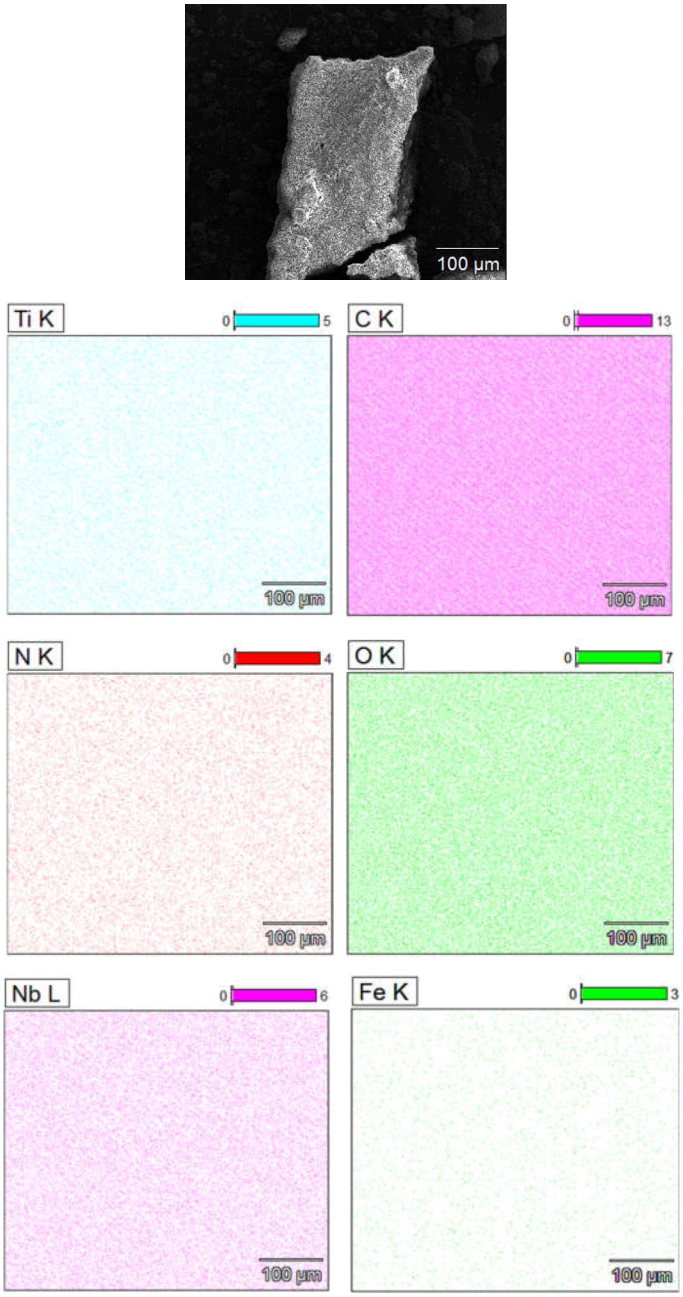

(d)

Figure S2. SEM elemental mapping of (a) NH_2-MIL-125(Ti), (b) $FeNbO_4$, (b) $FeNbO_4$ $_{(25\%)}$/NH_2-MIL-125(Ti) $_{(75\%)}$, (c) $FeNbO_4$ $_{(50\%)}$/NH_2-MIL-125(Ti) $_{(50\%)}$ and (d) $FeNbO_4$ $_{(75\%)}$/NH_2-MIL-125(Ti) $_{(25\%)}$.

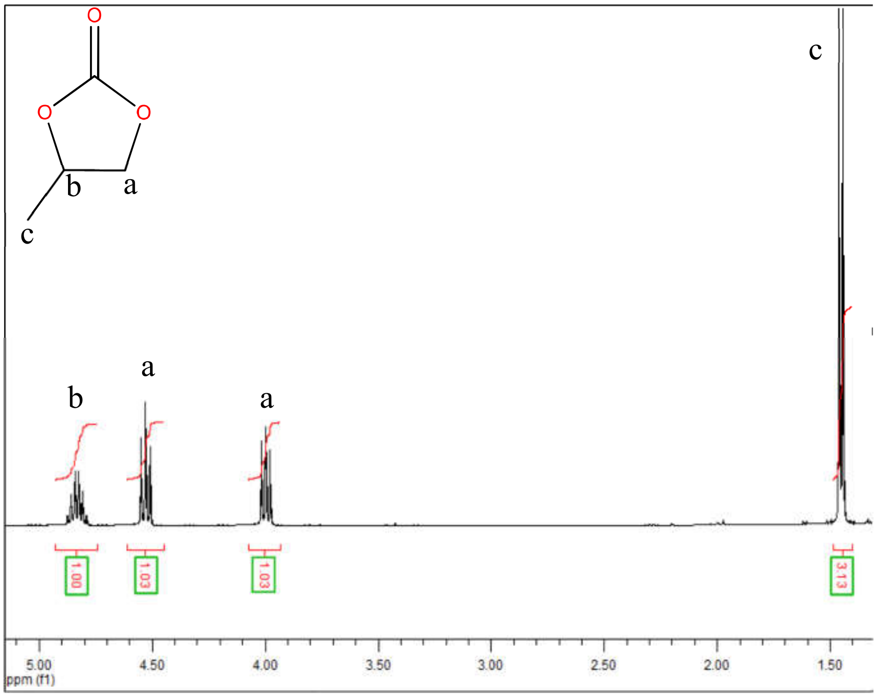

Figure S3. ^{1}H NMR spectrum for obtained propylene carbonate.

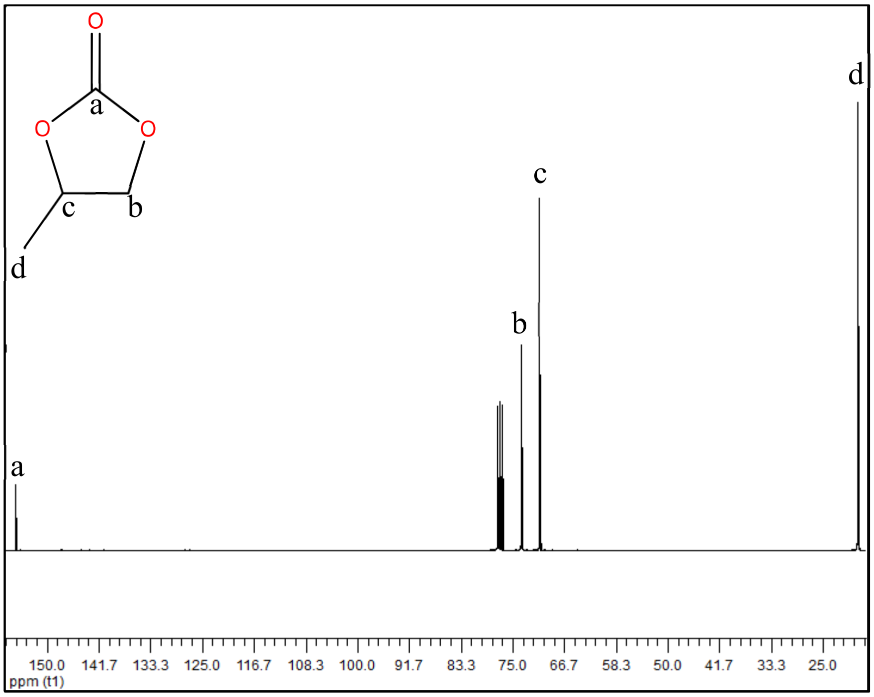

Figure S4. ^{13}C NMR spectrum for obtained propylene carbonate.

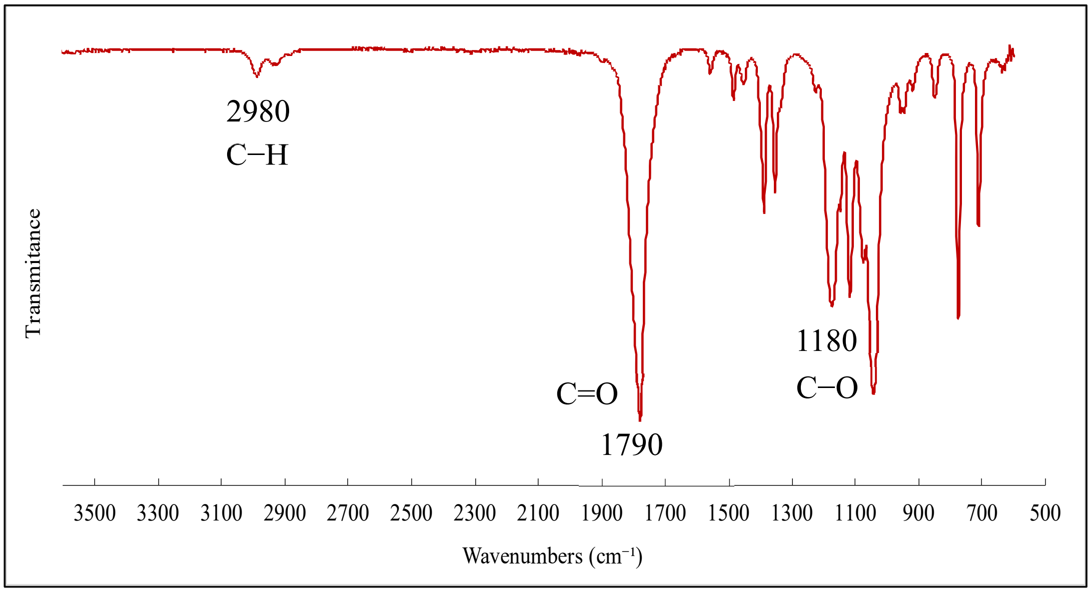

Figure S5. FTIR spectrum for obtained propylene carbonate.
